# Practical Points in the Treatment of Rheumatism

**Published:** 1903-09-05

**Authors:** 


### PRACTICAL POINTS IN THE TREATMENT OF RHEUMATISM.

The virtues of salicin and the salicylates in the treatment of rheumatic fever have been so generally recognised that the older methods of treatment are often entirely overlooked. Nevertheless, there were brave men before Agamemnon, and a parallel remark may be made in reference to the salicylates. There are still physicians who hold fast to the alkaline treatment of acute rheumatism, and without instituting any invidious comparisons it can be safely said that it is a therapeutic method of distinct value. A not infrequent cause of failure is the fact that the alkaline salts are not given in sufficiently large doses. To succeed, it is absolutely essential to order the potassium citrate, bicarbonate, etc., in such amounts as to produce and maintain an alkaline reaction in the urine. The doses necessary to effect this vary with the individual patient, but speaking generally, it will be found that doses considerably in excess of those commonly prescribed are needed to change the normal reaction of the urine. The test of sufficiency is readily applied, and the physician can never be sure that he is adopting the alkaline treatment in a thorough and efficient fashion unless he makes sure that his remedies are modifying the urine in the manner indicated. In many sub-acute cases the alkaline treatment, when fully practised, will be found to succeed when other well-known remedies have failed. It is easy to associate it with quinine when a tonic influence is required, and the best way is to add the quinine to each dose of the alkaline mixture at the time of administration. The quinine, of course, does not dissolve, and the patient is thus spared the bitter taste of the remedy. Chronic rheumatism, it is generally said, does not yield to the salicylates, and certainly the chronic, painful enlargements and deformities of the joints variously termed rheumatic or rheumatoid arthritis are undoubtedly very resistant to all forms of treatment. Yet sodium salicylate is not without virtue in this condition. Its special influence is to lessen the stiffness and discomfort which the patient experiences when he wakes in the morning. This is very often a cause of considerable disability, and it is in many instances much improved by sodium salicylate given in a single full dose at bed time. The patient may commence, say, with 15 or 20 grains, and this should be steadily increased until either relief is obtained or physiological evidences of the action of the salicylate are produced. The remedy can be continued for weeks or months and frequently with great advantage. A local application of great service in the same disease is a strong liniment of capsicum. This should be sprinkled on flannel which is to be wrapped round the affected joints and worn all night. Menthol is another remedy which often gives prompt relief to rheumatic pains, more especially when these affect the muscles. The best method of applying it is in the form of an ointment made with equal parts of lanoline and vaseline. The proportion of menthol ordered should be a drachm in an ounce of the ointment. The greasy basis permits the use of energetic friction and also favours the passage of the menthol through the epidermis.

